# Ocean Acidification Has Multiple Modes of Action on Bivalve Larvae

**DOI:** 10.1371/journal.pone.0128376

**Published:** 2015-06-10

**Authors:** George G. Waldbusser, Burke Hales, Chris J. Langdon, Brian A. Haley, Paul Schrader, Elizabeth L. Brunner, Matthew W. Gray, Cale A. Miller, Iria Gimenez, Greg Hutchinson

**Affiliations:** 1 College of Earth, Ocean, and Atmospheric Sciences, Oregon State University, Corvallis, OR, United States of America; 2 Coastal Oregon Marine Experimental Station and Department of Fisheries and Wildlife, Hatfield Marine Science Center, Oregon State University, Newport OR, United States of America; 3 Department of Fisheries and Wildlife, Oregon State University, 104 Nash Hall, Oregon State University, Corvallis, OR, United States of America; University of Western Sydney, AUSTRALIA

## Abstract

Ocean acidification (OA) is altering the chemistry of the world’s oceans at rates unparalleled in the past roughly 1 million years. Understanding the impacts of this rapid change in baseline carbonate chemistry on marine organisms needs a precise, mechanistic understanding of physiological responses to carbonate chemistry. Recent experimental work has shown shell development and growth in some bivalve larvae, have direct sensitivities to calcium carbonate saturation state that is not modulated through organismal acid-base chemistry. To understand different modes of action of OA on bivalve larvae, we experimentally tested how pH, P_CO2_, and saturation state independently affect shell growth and development, respiration rate, and initiation of feeding in *Mytilus californianus* embryos and larvae. We found, as documented in other bivalve larvae, that shell development and growth were affected by aragonite saturation state, and not by pH or P_CO2_. Respiration rate was elevated under very low pH (~7.4) with no change between pH of ~ 8.3 to ~7.8. Initiation of feeding appeared to be most sensitive to P_CO2_, and possibly minor response to pH under elevated P_CO2_. Although different components of physiology responded to different carbonate system variables, the inability to normally develop a shell due to lower saturation state precludes pH or P_CO2_ effects later in the life history. However, saturation state effects during early shell development will carry-over to later stages, where pH or P_CO2_ effects can compound OA effects on bivalve larvae. Our findings suggest OA may be a multi-stressor unto itself. Shell development and growth of the native mussel, *M*. *californianus*, was indistinguishable from the Mediterranean mussel, *Mytilus galloprovincialis*, collected from the southern U.S. Pacific coast, an area not subjected to seasonal upwelling. The concordance in responses suggests a fundamental OA bottleneck during development of the first shell material affected only by saturation state.

## Introduction

The uptake of carbon dioxide by ocean waters due to increasing atmospheric CO_2_ concentrations and resulting change in marine carbonate chemistry is called ocean acidification. During absorption and hydrolysis of CO_2_ by marine and estuarine waters a number of changes occur to the carbonate chemistry system: dissolved inorganic carbon increases, P_CO2_ increases, pH decreases, and the calcium carbonate saturation state decreases. These changes are well understood and can be predicted using well-documented thermodynamic constants. However, in a given body of water, the rate at which each parameter of the carbonate system changes relative to a given increase in P_CO2_ will vary with temperature, salinity, depth, and alkalinity. These changes are still predictable using the thermodynamic equations and dissociation constants for the carbonic acid system; however empirically-defined relationships from one setting, e.g. the oligorophic open ocean, cannot be simply extended to another, e.g. coastal and estuarine waters. Other drivers of carbonate chemistry, whether natural or anthropogenic in origin, can result in additive effects between regional and global drivers [1–6]. The result is that future conditions predicted for the global ocean are already occurring in coastal systems [7–9]. Many temperate coastal zones have large seasonal fluctuations in salinity (and thus alkalinity), and climate change effects are further predicted to alter watershed hydrology [10], and thus salinity in estuaries. Many temperate coastal zones are also highly productive (or even eutrophic), with seasonal and daily peaks in production-respiration cycles related to the timing of freshwater delivery, light, stratification, and temperature [11–13]. Subjected to this variability, reproduction in many marine invertebrates is timed to take advantage of the optimal conditions to help ensure success of the sensitive larval stages [14], [15]. The increasing concentration of atmospheric CO_2_ from fossil fuel combustion is contributing to a shifting baseline in coastal marine carbonate chemistry, while also altering regional climatologies that modulate salinity and temperature. In total, the resulting changes in coastal carbonate chemistry are complex in space and time. A significant challenge in OA research, therefore, is determining marine organismal responses to specific components of the carbonate chemistry system, and documenting the effects of each parameter on ontogenic development and physiology. How we interpret effects of ocean acidification on marine organisms depends on a thorough understanding of experimental conditions and integration of experimental results with documented and predicted changes to the habitats in which sensitive organisms reside [16]. Such an interpretation is not entirely possible with many of the currently used experimental methods.

The bulk of mechanistic studies of OA have focused on marine organisms’ ability (or lack thereof) to compensate internal acid-base status in response to decreasing pH in the external environment [17], [18]. However, it has recently been found that other effects of OA on organismal physiology may occur outside of the context of acid-base regulation, with larval bivalve development and growth dependent on saturation state independent of pH or P_CO2_ [19], [20]. Other recent studies on bivalve larvae have altered carbonate chemistry in similar ways to conclude carbonate ion concentration was the controlling factor (not saturation state) [21], [22]. However, in one case the lack of a proper control questions the rigor of this conclusion [21], [20], while the other provides no clear insight as to why a specific carbonate ion concentration would limit shell formation given all forms of dissolved inorganic carbon are used to produce shell [19], [22]. We have previously presented evidence for a direct saturation state sensitivity during formation of the earliest shell in bivalve larvae due to the rapid rate of shell formation, the greater exposure of the calcification surfaces to ambient water, and a limited energy budget until completion of that initial shell [19]. There is no *a priori* reason to assume that all aspects of organismal physiology can only be affected by regulation of internal acid-base balance, especially in organisms where calcifying interfaces can be exposed to the external environment (such as bivalve larvae or pteropods). Even in corals, where it was long held that the calcium carbonate precipitation was from fluid isolated from ambient waters by a transport-selective bio-membrane, recent work using tracers has shown that there is some direct exchange between this calcifying space and the external environment [23], [24]. Thus insight into organismal responses to OA, particularly in coastal environments, may be gained by understanding the balance of different modes of action of different carbonate chemistry variables across organismal life history stages.

Recent meta-analyses and reviews have generally used pH as the unifying variable to examine effects across the extensive literature on organism responses to ocean acidification, due to the importance of acid-base regulation and the pervasive lack of full carbonate-system constraint in the majority of experimental work [25]. Although there is no lack of evidence for ocean acidification impacts on marine mollusks [25], [26], interpreting those findings is challenging without proper context. In naturally higher alkalinity waters greater elevations in CO_2_ levels are needed to generate similar ΔpH as other systems, and will result in much smaller changes in aragonite saturation state (Ω_ar_). For example, in an experimental study in the Ria Formosa lagoon, Portugal, (where total alkalinity ~3500 μmol kg^-1^) Barros et al. [27], used P_CO2_ levels of 1380 μatm to generate a pH of ~ 0.4 relative to ambient; however, Ω_ar_ was 2.16, nearly double what would be found if such P_CO2_ values were imposed on seawater with more typical alkalinity (typically over 1000 μmol kg^-1^lower). These differences in carbonate system responses to CO_2_ make it increasingly difficult to compare across experimental findings without complete description of the carbonate system given recent evidence of direct saturation state sensitivity of marine bivalve larval development to OA [20]. We are not arguing against the value in synthesizing previous work; if we however have an incomplete understanding of mechanisms for sensitivity of marine organisms to ocean acidification, interpretation and inferential power from those syntheses may be limited.

In order to explore these possibly complex responses of organismal physiology to OA, we conducted an experiment on a common and native mytilid mussel found along the US Pacific coast, *Mytilus californianus*. Utilizing an experimental approach to independently alter P_CO2_ and saturation state (and pH pseudo-independently) we tested the effects of these three carbonate system components on shell development, growth, respiration rate, and feeding in this species. To our knowledge, there has been no previous work directly examining distinctly different biological processes to different carbonate chemistry variables as we present here. We evaluate these acute responses during the first 48+ hours following fertilization, and discuss the findings in relation to our fundamental understanding of organismal physiological responses to ocean acidification, interpreting experimental results, complex changes of carbonate chemistry in the coastal zone, and implications for bivalve populations in an increasingly acidifying ocean.

## Methods

**Fig 1:**
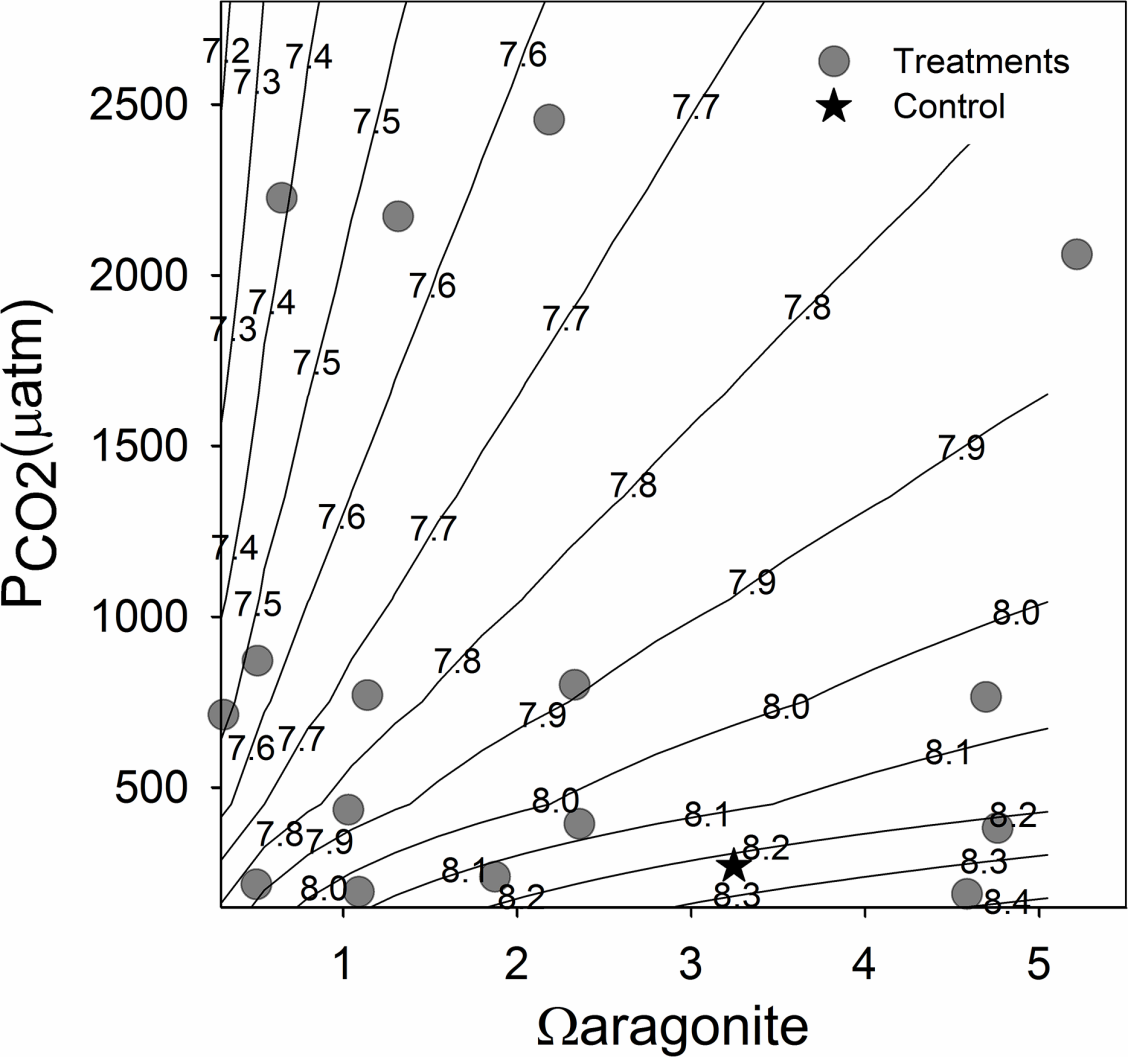
Experimental Carbonate Chemistry Treatments. Carbonate chemistry treatments of Ω_ar_ and P_CO2_ (μatm), with pH (total) isopleths to illustrate the relationship among the three variables in the experiments. Grey circles are the actual treatment values, and star indicates the treatment chemistry of the control (freshly collected seawater bubbled with CO_2_-reduced air for 24 hours).

We examined shell development, growth, respiration rate, approximately 48 hours post-fertilization in the California mussel, *Mytilus californianus*, in response to different carbonate chemistry system variables. We also examined initiation of feeding approximately 44 hours post fertilization. Employing a unique seawater chemistry manipulation framework we were able to completely separate effects of P_CO2_ and Ω_ar_, however pH was only pseudo-independent of the these two variables. The combinations of DIC and alkalinity that would be required to generate pH orthogonality within our current experimental range of P_CO2_ and Ω_ar_ are nearly impossible to obtain (see Fig 1 and isopleths of pH).

### 2.1 Larval rearing

Broodstock of *M*. *californianus* were collected from exposed inter-tidal rocks of Seal Rock on the mid-Oregon Coast. California mussels were collected under scientific collection permits 17066 and 17850 issued by the Oregon Department of Fish and Wildlife. The adults were held for 1–2 weeks in flowing ambient seawater (12 to 14^°^C; salinity 30–32 ppt) and fed *ad libitum* on a microalgal diet of *Isochrysis galbana* (Tahitian strain T-ISO) and *Chaetoceros* spp. Mussels were induced to spawn on August 19^th^ 2013 by increasing seawater temperature by 10^°^C over a period of about 1 h, followed by re-immersion in ambient seawater over 2–3 consecutive cycles. Addition of high concentrations of T-ISO further stimulated spawning. Females and males were transferred to separate 1-liter beakers once spawning was initiated. After gametes were collected from at least two males and two females, pooled eggs were fertilized by addition of pooled sperm in ambient seawater. Ten min after addition of sperm, the eggs were rinsed of excess sperm to prevent polyspermy [28].

Once polar bodies were observed within 2–3 hours of spawning, developing eggs were added to 500 ml biological oxygen demand (BOD) bottles at a density of 10 embryos per ml, with three replicate bottles allocated per seawater treatment. We did not apply the treatment during fertilization as trials found this to add to the total respiration in the BOD bottles from the presence of egg materials and un-successful eggs, thus creating a far greater bottle effect. The BOD bottles were stoppered, orientated on their sides and incubated for 48 hours at 18^°^C in the dark. At the end of the 48 h egg incubation period, three replicate subsamples were taken from each BOD bottle for determination of larval development and preserved in capped 20 ml vials by addition of 10% formalin buffered to ~8.1–8.2. Sub-samples of larvae were also sampled from BOD bottles for determination of respiration rates (see below).

### 2.2 Experimental design

We utilized a complete factorial design to determine the effects of P_CO2_ and saturation state (Ω_ar_) on development, shell size, and initiation of feeding. Four treatment levels were used for Ω_ar_ and P_CO2_ encompassing a range of Ω_ar_ from ~0.5 to 5.0 and of P_CO2_ from ~200 to 2500 μatm, resulting in 16 total treatments. Although we did not include a complete 3^rd^ factorial in our experiment for pH, we do have a range of pH values from ~ 7.4 to 8.4, with similar values across several P_CO2_ and Ω_ar_ levels (Table 1, Fig 1). Experimental seawater is treated and manipulated prior to the experiment in gas impermeable bags (Section 2.2), then that water was added to experimental chambers at the time of the experiment, as noted below. For larval shell metrics (development and growth), three replicate experimental containers per treatment (500 ml biological oxygen demand (BOD) bottles) were utilized, and 3 replicate counts per chamber were used and averaged for a mean per replicate prior to running statistics. Due to time constraints in measuring respiration rates, we used a reduced treatment design that included low and high P_CO2_ values at low and high Ω_ar_ values and two additional intermediate treatments (bold values in Table 1), resulting in six total carbonate chemistry treatments. For respiration rate measurements five replicate experimental containers were used per treatment (2 ml glass gas chromatography vials). In the initiation of feeding study, triplicate 20 ml containers were used per treatment.

**Table 1:** Complete Carbonate Chemistry. Carbonate chemistry of experimental treatments and control.

Ωtrt	CO_2_trt	Talk	DIC	HCO_3_	CO_3_	pH(total)	P_CO2_	Ωar
**Low**	**Low**	**749**	**679**	**639**	**31**	**7.84**	**219**	**0.50**
*Low	*MidLow	966	950	905	19	7.48	715	0.31
Low	MidHi	1367	1342	1278	32	7.54	873	0.51
**Low**	**High**	**2373**	**2393**	**2274**	**40**	**7.39**	**2228**	**0.65**
MidLow	Low	1095	965	889	69	8.03	197	1.09
**MidLow**	**MidLow**	**1446**	**1350**	**1269**	**65**	**7.84**	**437**	**1.03**
MidLow	MidHi	1977	1893	1792	73	7.75	773	1.17
MidLow	High	3378	3347	3188	82	7.55	2175	1.31
MidHi	Low	1614	1425	1297	119	8.11	241	1.87
MidHi	MidLow	2199	1996	1834	148	8.04	396	2.36
**MidHi**	**MidHi**	**2892**	**2723**	**2550**	**145**	**7.88**	**803**	**2.33**
MidHi	High	4754	4676	4451	137	7.64	2457	2.18
**High**	**Low**	**2497**	**2095**	**1797**	**290**	**8.35**	**191**	**4.58**
High	MidLow	3276	2894	2577	303	8.21	385	4.82
High	MidHi	4280	3943	3620	295	8.05	767	4.69
**High**	**High**	**6985**	**6686**	**6285**	**326**	**7.86**	**2063**	**5.21**
Control		2228	1945	1734	202	8.19	272	3.25

Experimental salinity and temperature were 31 and 18^°^C, respectively. Primary treatment levels for saturation state and P_CO2_ are noted in the left two columns. Total alkalinity (Talk), Dissolved inorganic carbon (DIC), Bicarbonate ion (HCO_3_), Carbonate ion (CO_3_) are all reported in μmol kg^-1^. P_CO2_ is in μatm. Treatment (trt) combinations in bold indicate those used for the respiration rate measurements. Asterisk denotes a preservation problem with this sample, and therefore P_CO2_ was greatly elevated relative to the initial treatment. The control was freshly seawater bubbled with CO_2_-reduced air for 24 hours.

The same cohort of larvae were utilized throughout the experiment, and all larvae were subjected to appropriate treatments prior to the experiments starting after the development and growth experiments (initiation of feeding and respiration). Developing embryos were stocked to triplicate BOD bottles 2–3 hours post-fertilization to ensure fertilization occurred and allow us to rinse fertilized eggs in order to prevent polyspermy. Larvae in the initiation of feeding were stocked into their 20 ml chambers at the same time as the developing embryos in the BOD bottles, and samples were taken at 44 hrs. post-fertilization. Larvae for the respiration rate measures were collected from their respective treatments at the termination of the development and size assay (48 hrs.) and stocked into respiration chambers within 3 hours following the breakdown of the development experiment.

We utilized two controls in the experiment: 1) seawater collected at the same time as the seawater collected for decarbonation (see Section 2.3), that was 1-μm filtered and stored at 2^°^C—to control for the effects of handling and chemical manipulations associated with the seawater treatments, and 2) 1-micron filtered seawater that was collected 24 h before the mussels were spawned and was then aerated with CO_2_-stripped (soda lime) air—to control for gamete quality and larval development in freshly collected seawater. Larvae in non-decarbonated seawater (control #1) were reared in BOD bottles set up in parallel with seawater treatments while larvae in fresh seawater (control #2) were reared in open 10 L polyethylene buckets that are commonly used for rearing bivalve larvae at HMSC [29]. Control data shown in the figures are for larvae reared in the freshly collected seawater.

### 2.3 Chemical manipulations

The complete details of our manipulation technique have been described elsewhere [20]; however an overview is provided here: seawater was pumped at high-tide to the Hatfield Marine Science Center (HMSC) from Yaquina Bay, Oregon, then filtered to 1-μm for chemical manipulation. An un-manipulated subsample was also filtered to 1 μm and stored at 2^°^C to control for possible effects of handling and processing the seawater; larvae developed just as well as in this water as in our fresh seawater control that was bubbled with CO_2_ stripped air. Trace metal grade HCl (34–37%) was added to the collected water at near alkalinity equivalence, and then it was bubbled with outside air for three days to remove most of the dissolved inorganic carbon (DIC). Next, the treated seawater was filtered to 0.22 μm, added to sterile 20 l polycarbonate carboys, pasteurized at 90^°^C for 3 h, cooled, then stored at 2–5^°^C until we were ready to complete the manipulations (in this case a couple days). At this time, the seawater was warmed to room temperature while aerating with filtered (0.22 μm) outside air to equilibrate P_CO2_ for at least 24 hrs. DIC and P_CO2_ were then determined for a discrete sample of the decarbonated seawater (DIC ~135 μmol kg-1) to determine DIC and total alkalinity to enable adjustment of carbonate chemistry parameters to target levels.

Target DIC and alkalinity concentrations were computed for 16 treatment combinations of P_CO2_ and Ω_ar_ under experimental conditions of temperature and salinity. Treatment combinations were generated by adding mineral acids and bases to the decarbonated seawater described above. To ensure accurate additions we added liquid reagents gravimetrically to customized 5 L gas-impermeable bags (EVOH-lined; Durashield^T^, scholle.com) adding first the DIC reagent to achieve target DIC concentration, then reagent grade, certified 0.1N HCl to adjust alkalinity. We created the liquid DIC reagent by adding NaHCO_3_ and Na_2_CO_3_ to deionized water in proportions to achieve an alkalinity of ~3.3 × 10^5^ μeq kg^-1^ and DIC ~2 × 10^5^ μmol kg^-1^, with an estimated P_CO2_ of 400 μatm, thus improving stability. Reagents were added rapidly to decarbonated water in the gas-impermeable bags while rapidly stirring with a magnetic stir-bar. Bags were immediately sealed with no head-space, and stored at 2–5^°^C for one week prior to the experiment, until water was used to fill experimental containers. Pilot studies indicated the carbonate chemistries of these seawater preparations were stable for over a one month period while stored in bags prior to experiments. Prior to stocking experimental chambers with larvae, the seawater bags were warmed to experimental temperature (18^°^C), 500 ml were added to each of three replicate BOD bottles per treatment, and antibiotics added (10 ppm ampicillin and 2 ppm chloramphenicol).

### 2.4 Carbonate chemistry measurements

Discrete samples were taken for carbonate chemistry at three time points: 1) following decarbonation but prior to manipulation, 2) immediately prior to filling BOD bottles from gas-impermeable bags, and 3) from each BOD bottle at the termination of the development experiment. Treatment data presented are from the samples in step #2 above. Seawater samples at the termination of the experiment were taken by placing a siphon tube into the BOD bottles, with its submerged end covered with a 37 micron Nitex screen to prevent removal of larvae. The siphon was started and the initial seawater flow discarded before sample collection with minimal aeration in a 350 ml amber glass bottle. All seawater samples collected in 350 ml amber glass bottles were preserved with 30 μl of saturated HgCl_2_, and sealed with polyurethane-lined metal crimp caps. P_CO2_ and DIC analyses were carried out via gas equilibration and stripping, respectively, followed by infrared detection, as in Bandstra et al. [30] and Hales et al. [31], modified for discrete samples. Standards for P_CO2_ and DIC encompassed the complete range of values in this study which are outside the range of typical modern-ocean seawater. To compute the complete carbonate chemistry we used, Millero [32] carbonic acid dissociation constants with temperature and salinity dependencies (which capture the Lueker et al. [33] seawater constants), Dickson [34] constants for boric acid, and Millero [35] water dissociation constants.

### 2.5 Larval responses

We evaluated four different larval responses to our carbonate chemistry treatments; development of prodissoconch I (PDI) shell, shell growth, respiration rate after 48 hours of treatment, and the proportion of feeding larvae at a pre-determined developmental stage (44 hrs. post fertilization).

#### 2.5.1 Development

At termination of the development experiment (48 hours post-fertilization) and after seawater samples had been taken, larvae were concentrated in a known volume of seawater and three replicate samples were collected from each BOD bottle and stored in 20 ml vials and preserved with buffered (pH 8.1–8.2) formalin. The average number of total larvae per replicate vial from each BOD bottle was 79 (±20), with a range from ~ 50 to ~100.

The proportion of normally developed larvae 48 hours post fertilization was evaluated under an inverted microscope, with normal development noted as a completely formed D-shaped shell with a straight hinge and the presence of tissue inside the shell. We previously found that velum extrusion was sometimes related to the speed and strength of additions of buffered formalin; therefore, we scored larvae as normal if larvae showed minor velum extrusion and normally developed shells. Normal shell development has been used previously as a water quality assay [36], [37] and in previous studies of OA effects on embryogenesis in bivalve larvae [38], [20].

#### 2.5.2 Shell growth

Shell size was determined by taking photographs of all sampled larvae scored as normally developed using an inverted transmission microscope (Jena Sedival 250-CL coupled to a FujiFilm Digital SLR S5) at 50x magnification and measuring shell lengths (longest axis parallel to shell hinge) on size calibrated images (ImageJ v 1.42). Shell lengths were measured for a total of 4639 normally developed individual larvae. We only measured size of normally developed larvae to prevent bias in the size estimates (poorly or undeveloped larvae are always smaller) and to prevent conflation of developmental and growth effects in interpreting results.

#### 2.5.3 Respiration rate

Respiration rates were measured for 48-h larvae that were first pooled from triplicate BOD bottles of each seawater treatment, after samples had been taken from each BOD bottle for determination of development and water chemistry analysis. Larvae were stocked at an estimated concentration of 500 larvae in each of five replicate 2 ml, solid-capped GC vials filled with the same seawater treatment as they had experienced during development in the BOD bottles. Larvae were concentrated and enumerated prior to stocking such that the larvae suspension constituted less than 10% of the volume added to each 2 ml vial. The desired concentration of 500 larvae per vial was chosen to yield the strongest and most consistent respiration signal while minimizing the effect of respiration on ambient carbonate chemistry within each treatment. Preliminary analyses showed that respiration rates under normal conditions were unaffected by larval densities between 200 and 800 larvae per vial (data not shown). Fluorescent oxygen-sensitive sensor spots (5 mm planar oxygen-sensitive spots, PSt3, PreSens, Germany) were pre-attached to the base of each vial before seawater or larvae were added. Oxygen measurements were made using the Fibox 3 (PreSens, Germany) that utilizes a fiber-optic cable to transmit and receive light from a sensor spot through the glass vial, allowing oxygen measurements to be taken in a non-destructive manner and without opening the vials. The vials were filled with warmed (18^°^C) seawater siphoned from seawater contained in the same impermeable bags as that used to fill the BOD bottles, followed with additions of chloramphenicol and 10 ppm ampicillin to control bacterial respiration. Five control vials (without additions of larvae) per seawater treatment allowed correction of larval respiration measurements for background bacterial respiration. Larval respiration rates were approximately 2–3 times those of background bacterial rates. Following larval additions, solid caps with PTFE liners were tightly screwed onto each vial, taking care to eliminate any bubbles from the vials. Vials were held on their sides submerged in a temperature-controlled seawater bath to maintain an experimental temperature of 18^°^C. Oxygen measurements on all 60 vials were taken every 2 hours over the first 6 hours of incubation (including time 0, initiation of respiration measurements), allowing linear regression of four time-points per treatment. We used a 6-hour incubation period because longer periods resulted in reduced respiration rates. The slopes of the regressions were corrected for background bacterial respiration determined in the control vials. Once respiration rate measurements were completed, larvae from each vial were preserved, counted and respiration rates expressed per larva (abnormal plus normal). The total average number of larvae per vial was 457 +/- 89 (1 standard deviation).

#### 2.5.4 Initiation of feeding

To assess the impact of water treatments on development of feeding organs and processes, we determined the proportion of larvae that ingested fluorescent beads at 44 hours post fertilization (initiation of feeding; IF). Fluorescent microspheres have shown to be an effective tool to monitor feeding activity in wide variety of invertebrates including bivalve larvae [39, 40], ciliates [41], freshwater and marine copepods [42], echinoderm larvae [43], mosquito larvae [44], and rotifers [45]. Unlike algal diets for bivalve larvae, fluorescent microspheres possess several useful qualities for measurement of larval feeding: 1) they are inert and will not react with experimental conditions; 2) they are uniform in shape size, size, and surface characteristics; 3) they are easy to count in translucent larvae under an epifluorescent microscope; 4) they do not need to be analyzed immediately and can be stored indefinitely. Preliminary experiments showed that at 44 hours post-fertilization ≥50% of *M*. *californianus* larvae began feeding when reared in natural seawater at 18^°^C. Fertilized eggs were stocked at 10 ml^-1^ in triplicate 20 ml solid-capped, sealed vials completely filled (no head space) with seawater treatments, at the same time as larvae were stocked in BOD bottles. After addition of fertilized eggs, 2μm Fluorescbrite yellow beads (excitation maxima at 441nm and emission maxima at 485nm; Polysciences Inc., Warrington, PA) were added to the vials at a concentration of 20 beads μl^-1^. Larval density effects, including the density used here, on microsphere availability were found to be negligible during preliminary experiments. Although these are higher than found in natural systems, for short-term, experimental comparisons our larval and bead densities did not appear to skew results. The bead size chosen for these experiments was based on findings of Baldwin and Newell [46] who determined a particle-size preference of 1–3μm for early oyster larvae (*Crassostrea virginica*). At 44 hour post-fertilization, the experiment was terminated by adding 40μl of 10% buffered formalin (pH ~8.1–8.2) to the vials. A minimum of 20 larvae per replicate vial were later examined under epifluorescent microscopy for the presence or absence of beads within their guts. The proportion of larvae feeding was then determined as the ratio of larvae that had at least 1 bead in their gut to the total # of larvae counted.

### 2.6 Data analyses

Our experimental design was a 4x4 factorial with Ω_ar_ and P_CO2_ as the primary factors, with four treatment levels of each. Our initial data analysis for all measured responses was a 2-way analysis of variance (ANOVA) followed by regression analyses to examine possible pH effects within treatment levels of the primary factors. Since we conducted the respiration experiments on a reduced suite of treatments, we carried out a two-way ANOVA on only the high and low levels of each primary factor (Ω_ar_ and P_CO2_), but ran pairwise comparisons on all treatments. For ANOVAs we calculated η^2^ (the total variance explained by each factor in the model) using sums of squares (SS) as SS_factor_/SS_total_. Possible pH effects were evaluated by regressing each physiological response to pH within each level of the primary factor found to be significant. For all data analyses assumptions of normality and equal variance were checked with visual inspection of data and Shapiro-Wilk’s test, and Levene’s test, respectively. Assumptions were met, unless otherwise noted. Non-linear and logistic regressions were run in SigmaPlot version 12.5. All other statistics were run using SAS Software version 9.3.

## Results

### 3.1 Chemistry data

**Fig 2:**
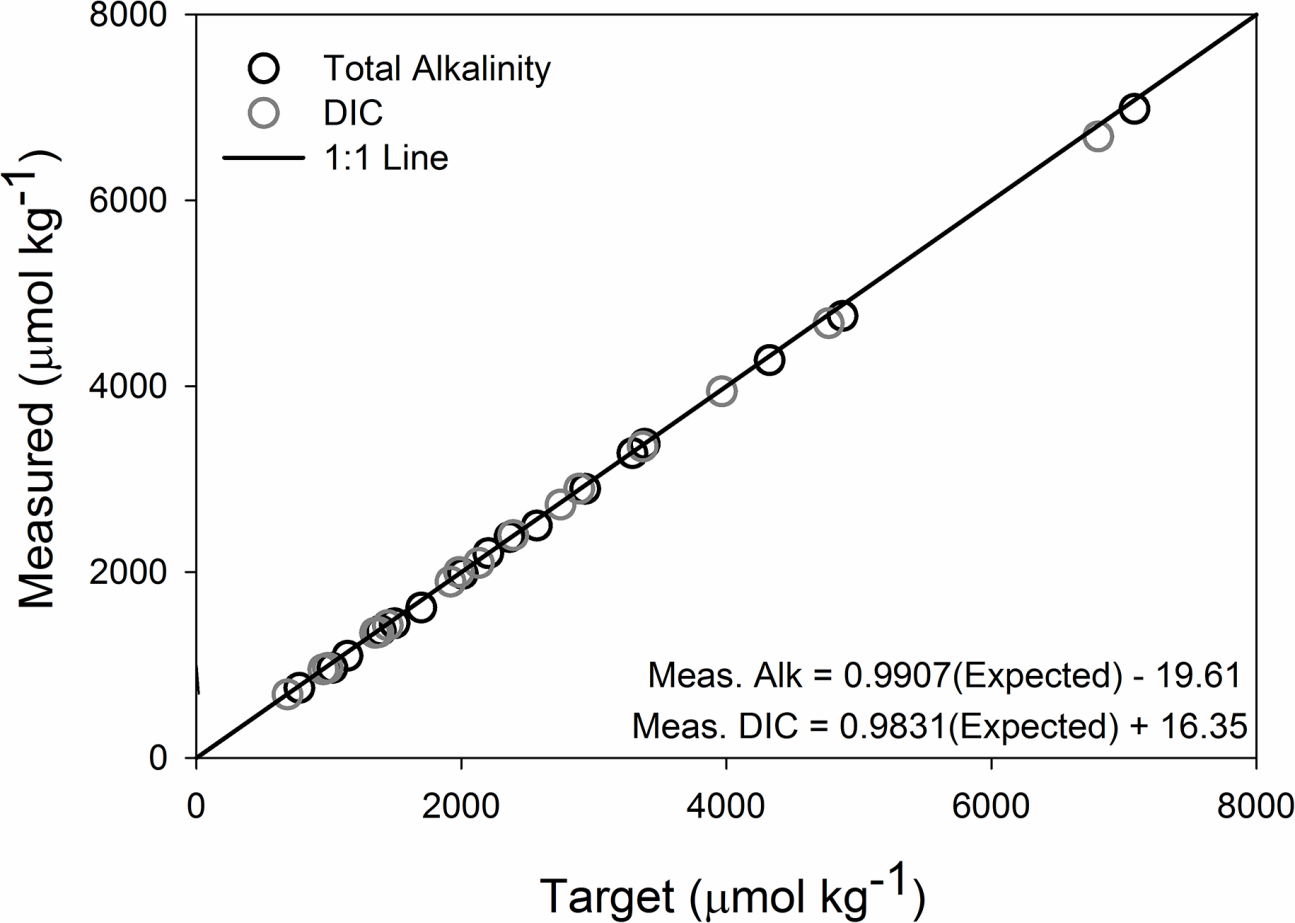
Target and Measured DIC and Total Alkalinity. The target and measured values of total alkalinity and total dissolved inorganic carbon (DIC) in μmol kg^-1^. The 1:1 line represents if our measured measured values were exactly aligned with the target values.

We were able to successfully create distinct treatment levels of P_CO2_ and Ω_ar_ by altering DIC and alkalinity concentrations (Figs 1 and 2 and Table 1). It is important to note that pH was pseudo-independent of the two primary factors in our design, with true independence within a subset of, but partial correlation over the entire, experimental matrix. Fig 1 shows pH isopleths within the treatment matrix. We did appear to have two treatments that deviated from our targets: the low Ω_ar_/mid-low P_CO2_, and the mid-hi Ω_ar_/low P_CO2_ treatments. In the first case, it looks as if there was a preservation issue, with P_CO2_ over 3x higher than the target, and a concurrent drop in Ω_ar_. In almost all of the treatments we see some lost alkalinity between what is measured and what is predicted based on our actual additions, typically 10’s of μmol kg^-1^. In earlier experiments [20] we found that during the addition of the liquid DIC reagent to the decarbonated seawater resulted in precipitation of calcium carbonate if the water was not stirred adequately. It seems most reasonable to assume we had some additional precipitation in the second case where our treatment deviated from the target, as it had the highest amount of missing alkalinity (82 μmol kg^-1^), and the P_CO2_ was more elevated than other low P_CO2_ treatments (excess of 41 μatm). We highlight these challenges for others who may want to take this experimental approach, as P_CO2_ is highly sensitive to small changes in the ratio of alkalinity to DIC (Table 1), even though our expected and measured DIC and total alkalinity values were in very close concordance (Fig 2).

### 3.2 Development

**Fig 3:**
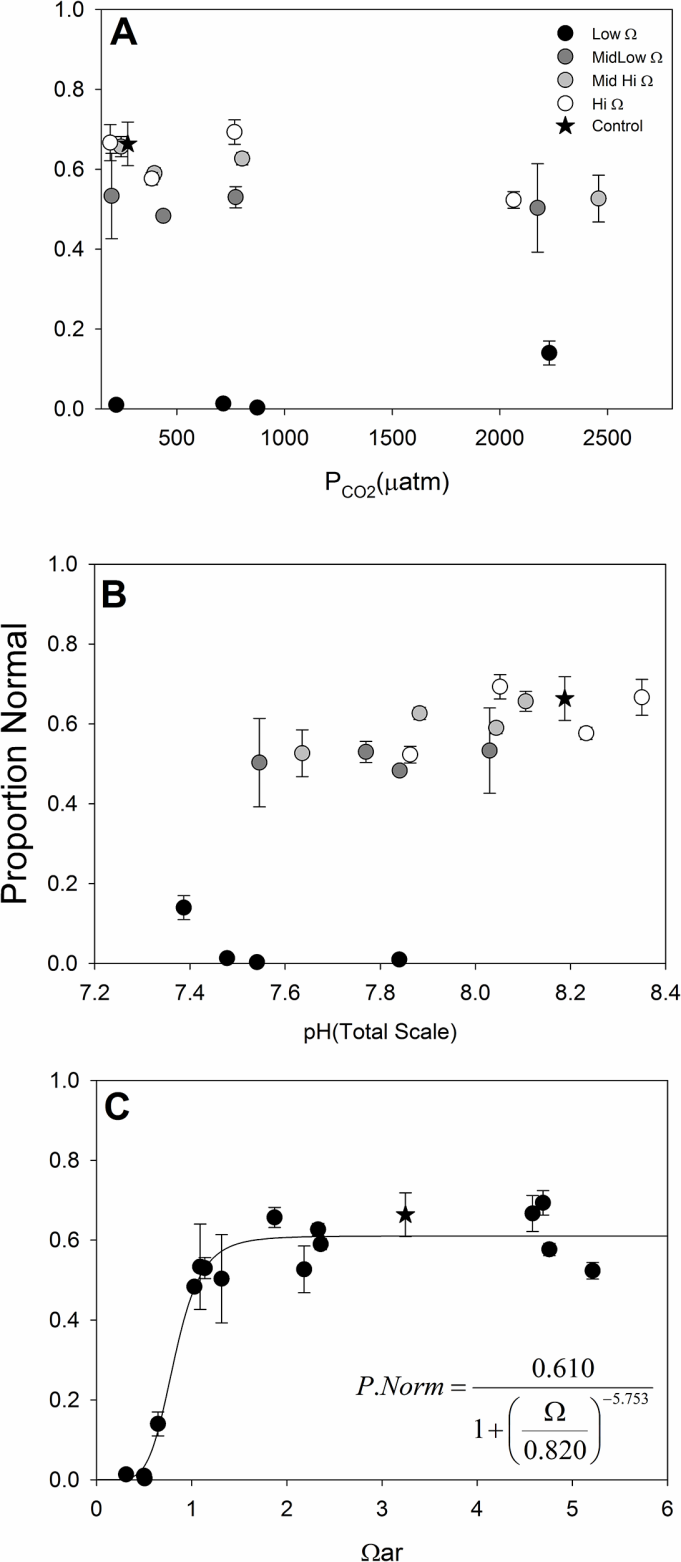
Proportion Normal Shell Development. The proportion of normally developed shells in relation to (A) P_CO2_, (B) pH, and (C) Ω_aragonite_. Greyscale symbols denote Ω_ar_ treatments in panels A and B, and the star is the control treatment. Error bars are standard deviations of the three replicate BOD bottles per treatment. See text for fit of model noted on panel C.

The clear and dominant effect of acidification on shell development in embryos of the California mussel, *M*. *californianus* was saturation state (Fig 3). We did however find statistically significant effects of saturation state (F_3,31_ = 529.80, P <0.0001) and the interaction of between Ω_ar_ and P_CO2_ (F_9,31_ = 11.59, P <0.0001), with no significant effect of P_CO2_ (F_3,40_ = 1.65, P = 0.1982) (Table 2). The significant interaction suggests caution in pairwise comparisons of treatment means, but the magnitude of each effect in the model implicates Ω_ar_ as the primary factor with it explaining 91.9% of the variance (Table 2). Given the large mean square for the Ω_ar_ effect (relative to the error and interaction terms), and the large error degrees of freedom (Table 2), it is likely the significant interaction term is a result of a Type I error (false positive). While the interaction term may be statistically significant, the lack of relevance is obvious upon visual inspection of the data (Fig 3). A problematic interaction term in a two-ANOVA is one in which the slopes of the Ω_ar_ treatment lines in Fig 3A cross, or are largely non-parallel. Given the lines are generally parallel in Fig 3A, the likelihood of Type I error, and that we are not testing differences among treatment means, the statistically significant interaction term may largely be ignored. A slightly positive effect of pH was found only in the Ω_ar_ = MH treatment (F_1,9_ = 11.74, p = 0.0075), with the slope of the linear fit on the back transformed data of 0.022 per 0.1 pH units. In other words, over the entire pH range in the MH treatment (~7.6–8.1) pH appeared to change percent normal by 11%. We therefore reiterate that saturation state had the principle effect on early shell development in this bivalve species. We fit a post-hoc three parameter logistic regression to the proportion normal response to Ω_ar_ to determine the functional response and found a highly significant fit (F_2,14_ = 167.42, Adj. R^2^ = 0.95, P < 0.0001; Fig 3).

**Table 2:** Shell Development ANOVA Table. Analysis of variance of shell development data in response to the primary factors in the experiment, Ω_ar_ and P_CO2_.

Source	DF	Sum of Squares	Mean Square	F Value	p-value	η^2^
**Ω**_**ar**_	3	4.5592	1.5187	592.80	<.0001	91.89
**P**_**CO2**_	3	0.0142	0.0047	1.65	0.1982	0.29
**Ω**_**ar**_ **x P**_**CO2**_	9	0.2991	0.0332	11.59	<.0001	6.03
**Residual**	31	0.0889	0.0029	.	.	1.79

The magnitude of the factor effect is estimated by η^2^, a measure of relative importance of parameters in the model calculated by SS_effect_/SS_total_.

Normal shell development was lower than what we observed for with other species in similar experiments [20]. Larval development in the control (filtered, CO_2_ stripped, fresh seawater) fell within the range of our upper Ω_ar_ treatment measurements, indicating that the lower development rates were not due to adverse effects of manipulation of the seawater chemistry but more likely due to the quality of the gametes. Supporting this conclusion, we observed similar lower developmental rates of eggs hatched in freshly prepared, non-manipulated seawater and those in our high omega treatments. There was one missing replicate in the mid-hi P_CO2_ and mid-hi Ω_ar_ treatments; however, the use of the mixed model procedure (Proc Mixed) in SAS (and the restricted estimated maximum likelihood techniques underlying it) for carrying out the statistical analysis prevented the missing observations from causing bias in the statistical results.

### 3.3 Shell Size

**Fig 4:**
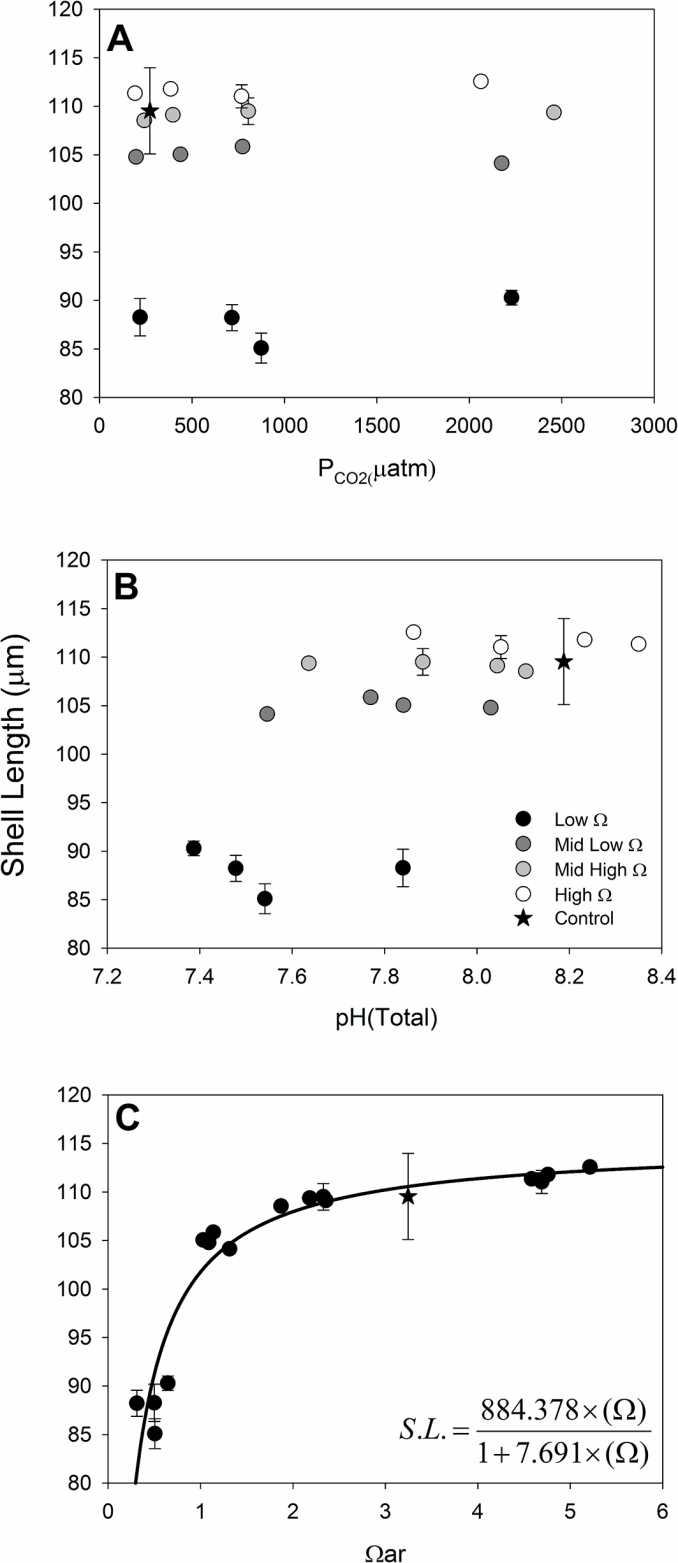
Normal Shell Length. Shell length of normally developed larvae (48 hours post fertilization and exposure) in relation to (A) P_CO2_, (B) pH, and (C) Ω_aragonite_. Greyscale symbols denote Ω_ar_ treatments in panels A and B, and the star indicates control data. Error bars are standard deviations of the three replicate BOD bottles per treatment. See text for fit of model noted on panel C.

Visual inspection of the data shows again a clear and dominant response of shell length to saturation state (Fig 4), and not P_CO2_ or pH. Statistically significant effects of saturation state (F_3,31_ = 1880.82, P <0.0001), P_CO2_ (F_3,31_ = 4.22, P = 0.013), and their interaction (F_9,31_ = 6.34, P <0.0001) were however found on shell length of normally developed larvae. The assumption of heteroscedascity was violated in the ANOVA and simple data transformations could not overcome the issue, which is driven largely by the greater variance around P_CO2_ treatments in the lowest saturation state treatment (Fig 4), with standard deviations in that treatment group being no more than 2% of the mean values. We therefore partitioned the saturation state treatments (the clearly dominant statistical effect, and the variable across which variance was unequal) into variance groups to define the covariance structure within the mixed procedure in SAS. ANOVA with a predefined covariance structure (based on variance groupings) and using restricted estimated maximum likelihood (REML) accounts for unequal variance among treatment groups. The new ANOVA returns nearly identical results with statistically significant effects of saturation state (F_3,10.9_ = 1145.02, P <0.0001), P_CO2_ (F_3,13.5_ = 4.50, P = 0.022), and their interaction (F_9,16_ = 6.83, P 0.0005). REML does not compute sums of squares, and therefore does not permit computation of effect size. Saturation state is however again the clear variable larvae are responding to for shell growth of normal shells (Fig 4), and in the original ANOVA it explained over 98% of the variance in the model. The very small error variance likely resulted in a Type I error (incorrect rejection of a true null hypothesis) with regards to the significance of the P_CO2_ and interaction effects. True effects of P_CO2_ or the interaction with Ω_ar_ would be clear in Fig 4; the parallel lines of shell length against P_CO2_ by Ω_ar_ treatment in Fig 4A also indicate no relevance of the interaction term, or P_CO2_ effect (Fig 4). Given the lack of any visual trend of pH effect within an omega treatment, we did not carry out linear regressions of pH effects. The fit of a hyperbola model for shell size against omega (Fig 4) resulted in a significant fit (F_1,15_ = 122.26, Adj. R^2^ = 0.88, P < 0.0001). All assumptions of the ANOVA and non-linear regression analyses were met.

### 3.4 Respiration Rate

**Fig 5:**
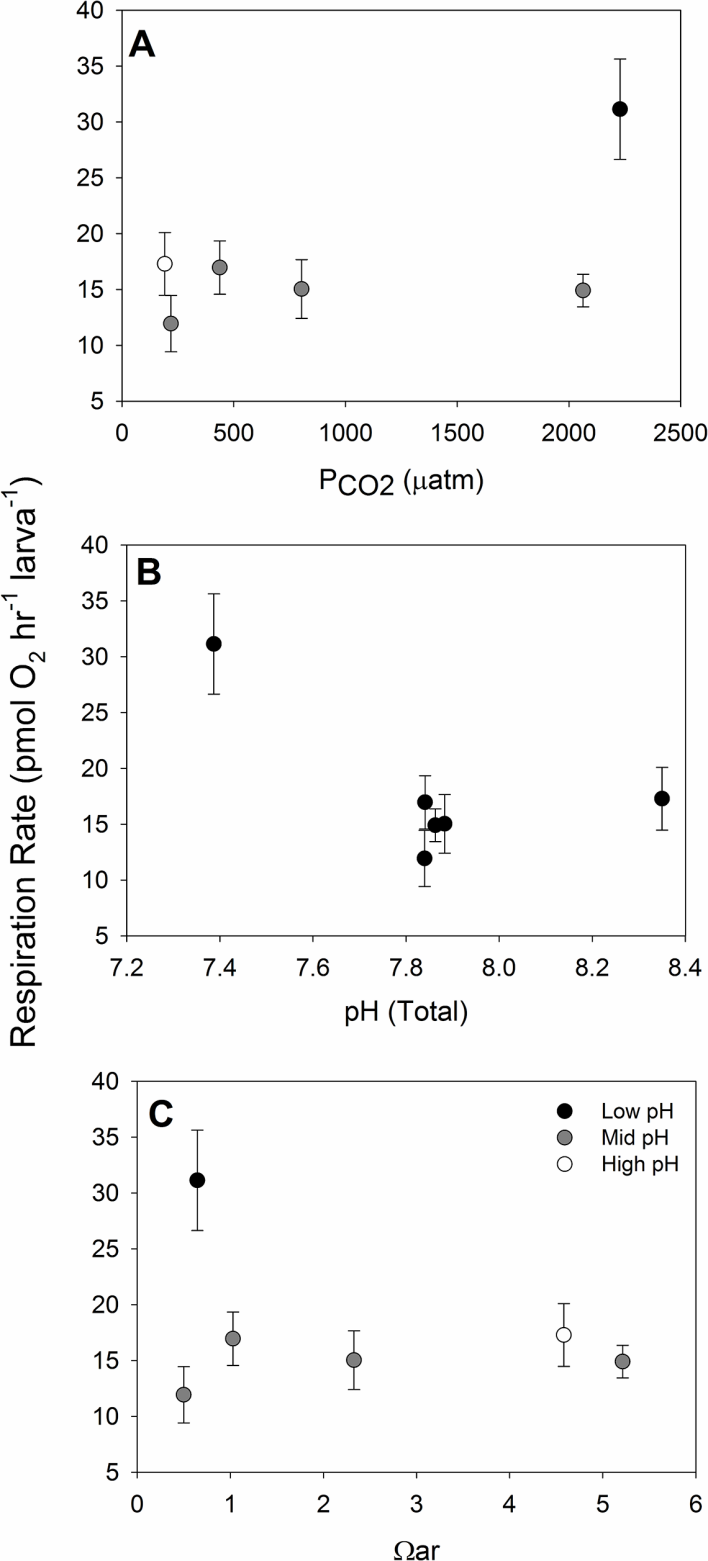
Larval Respiration. Respiration rate of larvae immediately following the 48 hour post-fertilization and experimental period in response to (A) P_CO2_, (B) pH, and (C) Ω_aragonite_. Error bars are standard deviations of the five replicate experimental chambers per treatment. The grayscale in this graph represents the three pH categories for the treatments used in the reduced experimental matrix (see Table 1).

**Fig 6:**
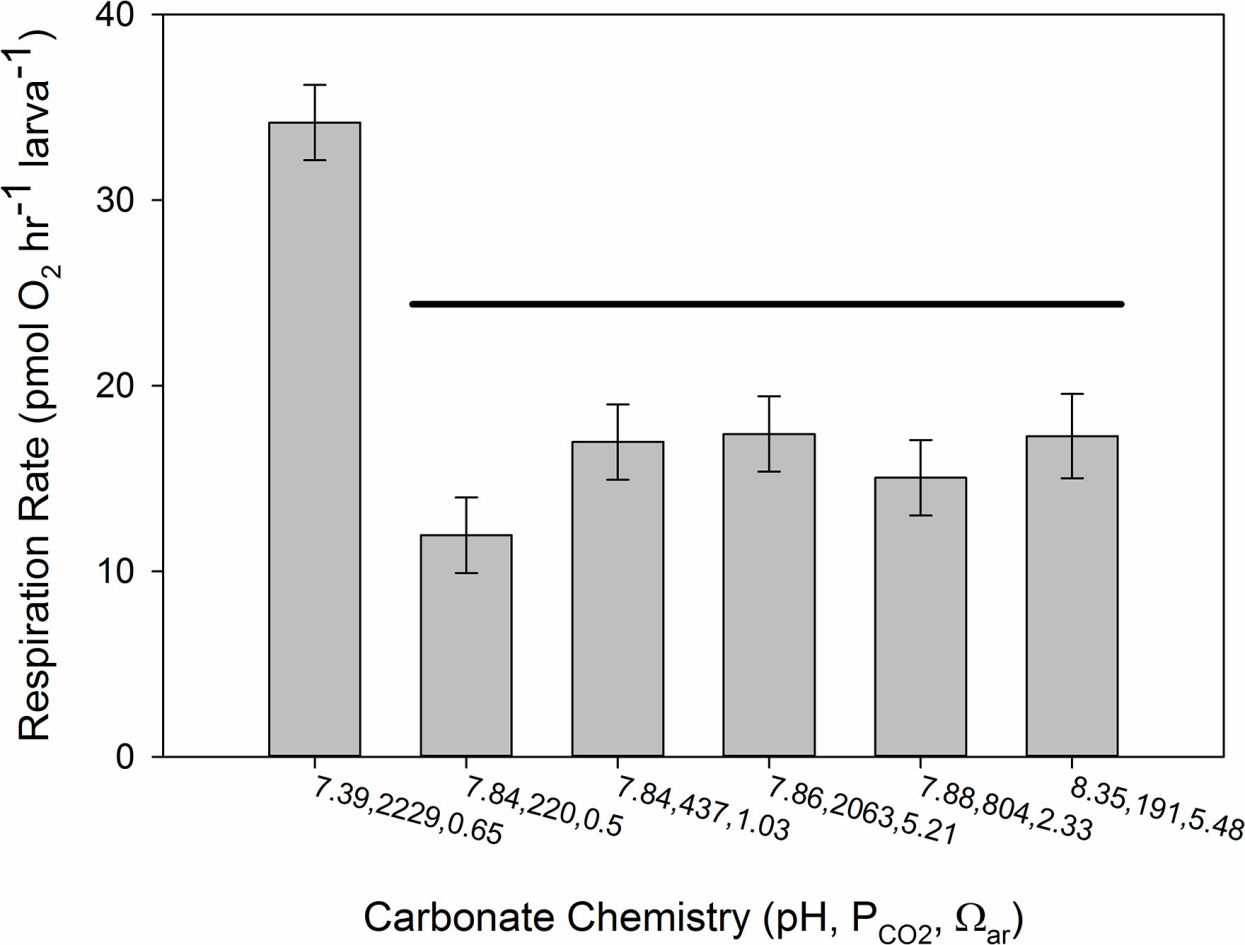
Comparisons Among Respiration Treatments. Respiration rates presented on a bar graph showing pairwise comparisons across all treatments, with the common line over values representing no significant difference. Error bars are standard errors. Note that values are arranged in increasing pH along the x-axis, and P_CO2_ and Ω_ar_ are included for each treatment. All values are in units previously noted.

In contrast to shell development and shell size, respiration rate was affected by pH and appeared insensitive to Ω_ar_ and P_CO2_ (Fig 5). We were unable to carry out respiration measurements on our entire suite of treatments, and thus picked a reduced treatment matrix to capture the high and low Ω_ar_ and P_CO2_ values and two intermediate values (bold values in Table 1). It is immediately apparent that respiration rate is sensitive to pH, but not as clearly to P_CO2_ and Ω_ar_ (Fig 5). However, the two way ANOVA indicated that P_CO2_ and Ω_ar_ had positive and negative statistically significant effects (Ω_ar_, F_1,15_ = 5.43, p = 0.0342; P_CO2_, F_1,15_ = 20.77, p = 0.0004), with a significant interaction (F_1,15_ = 20.35, p = 0.0004). Attribution of real effects due to P_CO2_ and Ω_ar_ would be incorrect as pH covaries with both parameters, and the response of respiration to the lowest pH value of 7.4 (Fig 5). The lack of direct P_CO2_ and Ω_ar_ effects are also evidence in Fig 6, where no statistical difference is seen between low and high P_CO2_ and Ω_ar_ at all pH values of ~7.8. Multiple pairwise comparisons (t-tests) of all treatments found that the high P_CO2_ low Ω_ar_ (lowest pH) treatment was significantly different than all the other treatments, with no other significant pairwise differences (Fig 6). Ideally, having multiple Ω_ar_ (or P_CO2_) conditions at our pH = 7.4 level (or pH = 8.4) would have provided more inferential power, but it becomes clear from Fig 1, that certain treatment combinations are not thermodynamically possible, and the inconsistent response of respiration to variable P_CO2_ and Ω_ar_ at pH = 7.8 further supports the role of pH above other variables on respiration rate.

When compared to the proportion normal and shell size data, there was no relationship between these two measures of shell properties and respiration rate, suggesting the response is not driven by shell developmental effects from the prior experimental exposure. In fact the highest and lowest respiration rates occur at Ω_ar_ = 0.50 and 0.65, where the proportions of normally developed larvae in the respiration chambers were only 0.22 and 0.24, respectively, and shell size was nearly identical (Fig 4). Therefore development or size differences cannot explain the differences in respiration rate.

### 3.5 Initiation of Feeding

**Fig 7:**
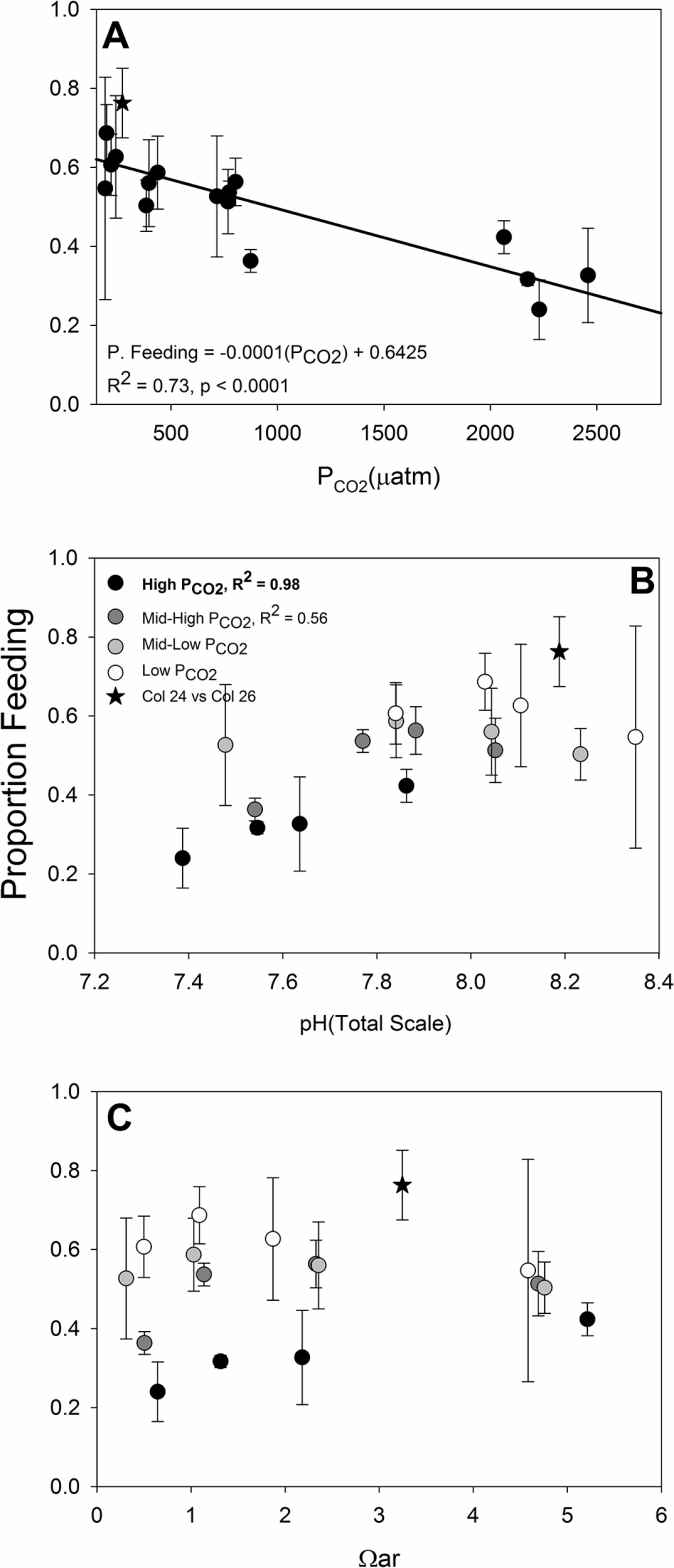
Initiation of Larval Feeding. The proportion of larvae feeding 44 hours post-fertilization and exposure in relation to (A) P_CO2_, (B) pH, and (C) Ω_aragonite_. Greyscale symbols denote P_CO2_ treatments in panels B and C. Error bars are standard deviations of the three replicate experimental containers per treatment. Bold value on panel B indicates the significant linear relationship between pH and initiation of feeding for the high P_CO2_ treatment. As noted in the text, the removal of this high P_CO2_ treatment results in extremely poor fit of the response to pH.

The number of larvae feeding at the completion of their D-hinge shell, 44 hours post-fertilization, was affected most by P_CO2_ (F_3,41_ = 15.05, p < 0.0001), and not by Ω_ar_ (F_3,44_ = 1.89, p = 0.1469), (Fig 7). The negative linear relationship between proportion feeding and P_CO2_ was significant, with fewer larvae feeding at elevated P_CO2_ (F_1,14_ = 43.73, R^2^ = 0.76, p < 0.0001). We found that pH also appeared to have a significant positive linear effect, with more larvae feeding at elevated pH (F_1,14_ = 11.66, R^2^ = 0.45, p = 0.0042), but the effect was driven entirely by positive effect of pH within the highest P_CO2_ treatment. A highly significant relationship was found between initiation of feeding and pH within the High P_CO2_ treatment (F_1,2_ = 108.83, R^2^ = 0.98, p = 0.0091); however if the High P_CO2_ treatment is removed from the overall regression and rerun, there is no longer a significant effect of pH on the proportion feeding (F_1,10_ = 1.76, R^2^ = 0.15, p = 0.5798). These findings highlight the significant effect of P_CO2_ on the proportion of larvae feeding, with a minor secondary effect of pH, but only under highly elevated P_CO2_.

## Discussion

In nearly all previous experimental studies of organismal responses to OA, pH and Ω_ar_ have been tightly coupled in response to alterations of P_CO2_. We utilized a unique experimental approach to determine the response of shell development, shell growth, respiration rate, and initiation of feeding to independent changes in Ω_ar_, P_CO2_, and pseudo-independent changes in pH. Thermodynamic constraints prevent some combinations of carbonate chemistry, such as our lowest pH (7.4) at the highest Ω_ar_ (~5), however the four levels of our two primary factors permitted multiple pH levels from ~7.5 to 8.2. Our results indicate there are multiple possible modes of action of ocean acidification, thus acid-base regulation cannot explain the suite of responses measured here and previously [3], [20], [21], [22]. The biological constraints bivalve larvae face under OA are: 1) rapid calcification during formation of the first shell [19], [20], [47], [48], 2) greater exposure of this calcification interface to the ambient environment [19] because calcification proceeds before completion of the PDI periostracum [49], [50], 3) reliance primarily on endogenous energy until completion of the PDI shell and feeding organs [19], [51] and 4) apparent limited ability to regulate acid-base status of internal fluids [18]. Shell development and growth in this native species of blue mussel, *Mytilus californianus*, responded similarly as two other bivalve species found in the US Pacific Northwest, that are non-native to the region, *Mytilus galloprovincialis* and *Crassostrea gigas* [20]. As with the other species, PDI shell development and growth of *M*. *californianus* were affected by saturation state, but not P_CO2_ or pH. However, in this study with *M*. *californianus*, we show that respiration rate and initiation of feeding did not respond to saturation state. Respiration rate was elevated in response to very low pH (but not saturation state or P_CO2_). Furthermore, the proportion of larvae feeding after 44-h post-fertilization was impacted by P_CO2_, and pH at high P_CO2_, but not saturation state.

Since the bulk of mechanistic studies of organismal responses to OA have focused on acid-base regulation within bodily compartments [17], it is important to note anatomical limitations of bivalves to “compartmentalize” the calcification process. Adult marine bivalves calcify from the extrapallial fluid (EPF), a semi-enclosed fluid between an organism’s body and its shell, the fluid in the outer-most space exchanges with seawater to varying degrees across taxa [52], [53]. As reviewed in Suzuki and Nagasawa [54], conflicting evidence suggests that Ca^2+^ ions are derived from seawater, across epithelial surfaces through the body [55]. Conversely, the carbon in the carbonate comes both from seawater DIC and respiratory carbon, with some leakage of DIC into the EPF from surrounding seawater [56]. While Carre et al. [57] showed that active transport of Ca^2+^ to the calcification site was not energetically possible at rates needed to support the observed rates of calcification; they argued that ion channels must readily allow Ca^2+^ ions to move rapidly across the mantle tissue to the EPF. Interestingly, bacteriological studies of EPF in adult clams indicate that the EPF is a “pseudo-internal” compartment that does have some exchange, but is also somewhat characteristic of hemolymph [58]. In adult bivalves, dependent on taxa and thus anatomy, many species do appear to have some direct exchange between the EPF and ambient environment [52]. In the early developmental stages of larvae, even less is known regarding the details of calcification of the first shell, PDI. It has been documented that calcification is initiated before the completion of the proto-periostracum called the pellicle [49], [50], [59], [60]. Even though there is a rapid increase in respiration during PDI shell formation [61], recent isotopic evidence of greater ambient-water DIC incorporation into the PDI shell [19] illustrates that the calcification surfaces are more exposed to the ambient environment. It therefore follows that calcification of the PDI shell should be more sensitive to changes in ambient carbonate chemistry. Recent experimental work documented a direct saturation state sensitivity, regardless of pH, of larval PDI shell development and growth in two distinct taxa [20], further supporting the exposure of PDI calcification to ambient chemistry, and thus the importance of saturation state to this larval stage. However, this does not preclude the impact of OA on other components of physiology; aspects that may be moderated directly through internal acid-base regulation [17], or manifest through other modes of action.

These results provide two important insights. First, they contribute more evidence in support of a kinetic-energetic hypothesis for a direct larval bivalve sensitivity to saturation state during PDI shell formation [19], [20]. Due to previously documented biological thresholds for bivalve larvae at Ω_ar_ > 1 [20], [62], and the relative sensitivity of saturation state to increasing P_CO2_ compared to pH, it becomes evident why a kinetic-energetic mechanism for larval bivalve sensitivity to Ω_ar_ is important. Failure of larvae to develop past PDI will preclude the importance of any stress (or energetic expense) associated with increased respiration at lower pH. Second, there is no one single carbonate system variable responsible for all organismal responses to OA, nor can these finding be explained solely through acid-base regulation. In other words, different components of larval physiology are affected by different carbonate system parameters for the species (and perhaps life-stage) studied. However, it is again, important to note that failure to develop past PDI represents a significant bottleneck in population dynamics, and while other carbonate system parameters may act as stressors, saturation state appears to matter most first for the rapid shell building PDI phase in bivalve larvae. That is not to say pH or P_CO2_ will not have other effects on later stages of bivalve larvae, we however lack direct experimental evidence. Other experiments designed to examine salinity as a co-factor in OA studies suggests saturation state may still be important for juvenile bivalve stages [3], [22], but this should be an area of future research with carefully controlled experiments. In open-ocean marine waters, over short geologic time scales, and in typical CO_2_-bubbled experiments, the carbonate system variables are tightly coupled. However, in coastal zones and over longer geologic time scales, the carbonate system variables can decouple [16], [63] as alkalinity and DIC deviate significantly from the values typical for the modern-day open ocean. Therefore, one challenge in looking ahead towards a more holistic understanding of OA impacts on organisms and ecosystems is to better understand the interaction of various drivers of coastal zone OA such as CO_2_ fluctuations in concordance or discordance with alkalinity fluctuations. A second, no less formidable challenge is to develop bioenergetic models that can incorporate multi-carbonate system stressors. We have demonstrated in this study that OA may act as a multi-stressor on bivalve larvae, with the multiple stressors residing only in the changes to the carbonate system. Although we note the importance of future OA work to include other potential stressors related to global change, such as salinity and temperature, our findings also suggest the current fundamental understanding of biological responses to OA in isolation is still incomplete.

In marine bivalves, other earlier studies have either directly or indirectly shown a saturation state sensitivity, however our current study is the first to show differential sensitivity of different physiological processes to different carbonate system parameters on the same species. In other words, the responses we found indicate that shell formation and growth, and to a lesser degree timing of feeding, are not responding to changes in internal acid-base chemistry. Prior studies have been suggestive of this; particularly shell formation and growth [3], [20], [21], [22]. The high demand for shell material combined with less-effective organismal control of EPF chemistry during early larval development results in greater sensitivity to decreasing saturation state due to the increased energy needed to sustain rapid calcification. It is critical to note that this appears independent of internal acid-base regulation. However, at slower calcification rates or at times during organismal life history when somatic growth may slow (or the organism has capacity to slow growth), the impact of this mechanism for Ω_ar_ sensitivity should be less significant. Since many bivalve larvae go through a period of very rapid calcification during the PDI shell period, this mechanism appears to provide the reason for the enhanced sensitivity in bivalve larvae to OA during the first couple days of life. It seems that an important PDI tradeoff must therefore be rapid shell development (at an energetic premium), providing capacity for more effective food capture versus slower development. However slower development at PDI results in increased duration of reliance on primarily maternal energy stores; thus some optimal balance must exist to complete PDI without depleting the limited maternal energy pool. Smaller size would also generally indicate longer larval period, thus increasing predation pressure on the population [64], [65]. In other words, even sub-lethal exposure to moderate OA during PDI will lower the scope for growth and result in a delay for larvae reaching a suitable metamorphosis size [62],[66], [67], [68]. Continued pH stress on the larval energy budget would likely compound the carryover effect from a saturation state impact during PDI shell formation, and further delay settlement. Should larvae fail to transition past PDI however, other carbonate system effects are mute issues. Conversely, sufficient energy stores and shorter larval duration both directly increase the probability of settlement success [69–74], and thus the impact of sub-lethal acidification stress on bivalve larvae should not be underestimated (as in [62], [75]). However, this kinetic-energetic based mechanism for sensitivity in marine bivalve larvae does not occur in isolation of other physiological processes.

Measuring respiration rates on larvae provide several challenges, however our rates were within the range of previously reported values for marine bivalve larvae [47], [51], [76], [77]. We measured respiration on already fully-formed larvae that had been exposed to the treatment conditions from immediately after fertilization, with a reduced treatment matrix. Previous work by Sprung [47] in *M*. *edulis* found that the mass specific metabolic rate (MSMR) increased nearly 4 fold from fertilization to soon after PDI (~ 48 hours), followed by an exponential decrease in MSMR through the rest of the life history, and thus our measures provide a snapshot of the entirety of larval respiration during the critical PDI development. Larval respiration rate in our experiments responded to pH, but not Ω_ar_ or P_CO2_, with a 2–3 fold increase in respiration at a pH of ~7.4, relative to those observed at ~7.8 and 8.3. It is however important to note that we lacked low and high pH treatments at different P_CO2_ and Ω_ar_ conditions due to logistical challenges in the experiment, and thermodynamic constraints on the carbonate chemistry; some combinations of carbonate chemistry are simply not feasible to produce. We do not believe this precludes our interpretation of the results, which are consistent with what would be expected. It is important to note that at a pH of roughly 7.8, there was not difference in respiration across a 10-fold difference in P_CO2_, and across a range of Ω_ar_ from 0.5 to 5.2. Examining the larval respiratory response to pH and size response to Ω_ar_ further suggests the energetic cost of making shell under acidification (a lower scope for growth) is not related to respiratory alterations of the energy budget, as may be expected under direct effects of OA on acid-base chemistry. We believe this provides additional critical support for a kinetic-energetic mechanism for the direct larval sensitivity to saturation state (as in [19], [20]).

Respiration rate in our study increased in response to lower pH, rather than exhibiting metabolic depression as some studies have found [78], [79], however CO_2_ concentrations in those studies were roughly 10,000 and 5,000 μatm CO_2_, respectively. Across a more moderate range of CO_2_ concentrations Thomsen and Melzner [80] found a response in *Mytilus edilus* adults similar to our observations, with an increase in oxygen consumption with decreasing pH (to ~ 7.4–7.5), and only a slight metabolic depression at their lowest pH of 7.14. As noted by Thomsen and Melzner [80], respiratory responses of marine organisms to decreased pH are variable and appear to be related to the ability to compensate internal acid-base via bicarbonate accumulation. However, the respiratory response and impact on energy budget is generally acknowledged as the primary mechanism for organismal responses to OA [17], [81], [82]. At least for some PDI bivalve larvae, we would argue that this is not the primary mechanism for acute and carryover effects during the early larval stage. However, we acknowledge these respiratory effects will contribute to a more holistic and integrative bioenergetics equation that can better predict organismal responses to OA. Maternal investment into eggs will ultimately be an important component of such an energy budget; we lack direct measures of egg lipid values from these experiments which can vary widely in mytilid eggs [74], [83], [84].

With a limited and diminishing energy supply until completion of the PDI shell and development of feeding organs, the timing of the onset of feeding is an important threshold for larvae to begin rebuilding energy stores for growth and metamorphosis. Although we did not directly determine the timing for the onset of feeding of *M*. *californianus* larvae in our experiments, we measured the proportion feeding at a specific developmental time point previously determined to include a significant portion of feeding larvae under normal culture conditions. Interestingly, we found that counter to both shell development and growth as well as respiration, that initiation of feeding was most affected by P_CO2_, with smaller effects of pH at elevated P_CO2_. The magnitude of the response was not very large, with a difference of about 20% in the proportion of feeding larvae between low and high P_CO2_ treatments. The lack an effect of saturation state suggests this is not directly related to development of the shell and attachment of the velum, the larval feeding appendage [85]. Others have previously documented uncoupling between tissue and shell growth for older *M*. *californianus* larvae under OA stress [86], in adults of other bivalve species[87–89], and in adult urchins under OA stress [90]. If different carbonate system parameters are affecting development of hard and soft parts differently, P_CO2_ could have stunted the development of feeding organs, resulting in a delay in the effective particle capture and ingestion by larvae. Reduced development rate and delayed onset of behaviors has been observed among several invertebrate species held in elevated CO_2_ environments [91–94]; however it is unclear how CO_2_ may be generating these ephemeral effects.

In contrast to H^+^ ions, carbon dioxide can diffuse across biological membranes [95] and affect intracellular pH. Intracellular pH is known to affect a wide range of cellular processes [96]. It is unknown if such changes in intracellular pH and P_CO2_ could affect development or functioning of larval feeding organs, such as the ciliated velum (Newell and Langdon 1996); however, Schmid et al. [97] reported that elevated intracellular CO_2_/HCO_3_^-^, independently of pH, can directly affect beat frequency of cilia of the epithelium lining human airways. Furthermore, nerves controlling gill function may also be affected by reduced intracellular pH because high [H^+^] negatively impacts gap junction channel permeability, reducing invertebrate synapse performance [98], [99]. Although delays in energy acquisition through feeding are known to inhibit subsequent growth and development of planktotrophic larvae and impose constraints on later life stages [100], [101], it is presently unclear if the observed delay in the onset of feeding of early *M*. *californianus* larvae could significantly affect subsequent larval growth and development. Nonetheless, our results suggest feeding is another key physiological process affected by ocean acidification. This finding supports recent studies which observed negative impacts of acidified seawater on the feeding physiology of marine planktotrophic invertebrate larvae, including selection efficiency [102], gut size [103], and digestion [104].

Taken individually the responses of shell development and growth, respiration, and initiation of feeding to Ω_ar_, pH, and P_CO2_, respectively, provides interesting insight, and challenge, to predicting organismal responses to ocean acidification. Of critical importance is the recognition that the mode of action of ocean acidification extends beyond only internal acid-base regulation. Each of our measured responses will alter the larval energy budget, and in coastal systems, Ω_ar_, pH, and P_CO2_ can change in less predictable ways [2], [16]. A more integrative and comprehensive approach is clearly needed to better anticipate ocean acidification effects on marine organisms, particularly in coastal habitats. This appears to be especially true for organisms that have life-history periods with rapid shell production, where calcification surfaces may be more exposed to environmental conditions, as in early developing bivalve larvae [19]. A better-integrated approach to understanding organismal responses to OA should also make comparisons among species more tractable, which will prove important in trying to identify traits that may make some species more susceptible to OA stress, such as ability to regulate internal pH [81], [82], or demand for rapid shell formation [19].

**Fig 8:**
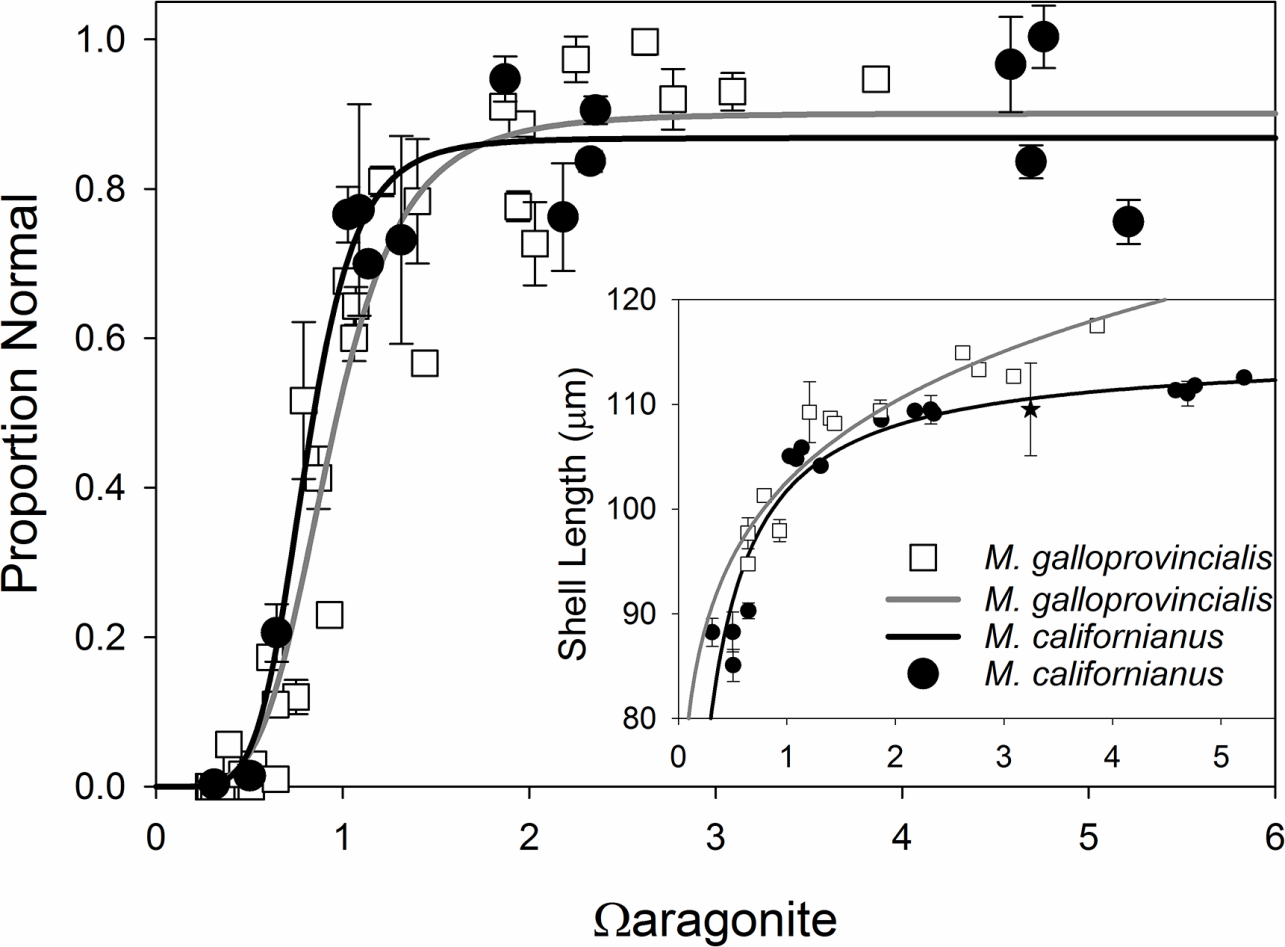
Reponses of Two Mussel Species to OA. Comparison of normal shell development and shell length (insert) of normal larvae between *M*. *californianus* (black circles, this study) and *M*. *galloprovincialis* (open squares, from previous work by our group [20]). Error bars for both studies are standard deviations. Proportion normal data for *M*. *californianus* is been standardized to the control values make the data more comparable with the *M*. *galloprovincialis* study.

Our results and experimental techniques in this study allow us to directly compare two mytilid species found in Oregon’s coastal waters: the native California mussel, *M*. *californianus* (this study), and the non-native Mediterranean mussel, *M*. *galloprovincialis* (data from [20]). When plotting control-normalized proportion of normally developed shell and normal shell size against Ω_ar_, we see virtually identical responses to Ω_ar_ by both species (Fig 8). This congruity in functional response raises some interesting questions. First a hypothesis in the OA literature is that organisms in more variable or higher CO_2_ environments will be pre-adapted and more resilient to ocean acidification [105–110]. The Mediterranean mussel broodstock used in previous experiments [20] were obtained from Carlsbad Aquafarm located on Agua Hedionda Lagoon, CA, where the stock has been farmed for roughly 20 years and shows no indication of hybridization with other mytilids based on morphological characteristics (Mark Smith, manager, pers. comm) or genetic analyses [111]. The outer lagoon, where the mussel farm is located, is well flushed by the Pacific Ocean and this coastal region does not experience the significant seasonal upwelling found along the central Oregon coast where the *M*. *californianus* used in this work were collected. While we lack data from the specific locations where the broodstock were collected previous surveys along the U.S. Pacific coast generally show more corrosive waters further north [112–114]. We would anticipate that if selection for traits leading to adaptation was occurring, California mussels from the Oregon coast would be more resilient to acidification, given their exposure to elevated CO_2_ associated with upwelling along the Oregon coast [7], [8], [62], [112]. Therefore, it is curious to see similar responses between these two species originating from such distinct OA environments. One would expect that larvae of *M*. *californianus* would be exposed to strong selective pressure as adult *M*. *californianus* spawn throughout the summer [115] when upwelling events typically occur on the Oregon coast. At this early developmental stage, it is plausible that constraints on the biological and physio-chemical processes of shell formation allow little adaptive capacity to achieve rapid shell formation under OA stress. Two traits that could provide resiliency would be egg size or shell formation rate. Maternal bivalve investment in eggs is often a balance between increased energy per egg (which should increase individual survival) and decreased number of eggs that lowers the probability of cohort survival [116–118]. Secondly, larvae developing more slowly through the PDI stage should overcome this direct saturation state stressor; however, with a slower developing shell, larvae would need to balance this energetic gain against a longer developmental time and higher respiratory losses before capture of particulate food sources was possible.

Although we have made great strides in understanding the consequences of ocean acidification on marine organisms, our results highlight some exciting new avenues for research and shed significant insight into why bivalve larvae are so sensitive to ocean acidification. First and foremost, our results highlight the real challenge in interpreting organismal responses to OA without knowledge of the complete carbonate chemistry, and an understanding of the mechanisms for positive, neutral, or negative responses. Many larval bivalve exposure experiments to date have only applied treatments following development of the PDI shell, as significant challenges exist in either spawning bivalves or handling developing embryos from commercial hatcheries. Second, as alkalinity varies significantly across regions and in estuaries, simply interpreting experiments on CO_2_ or pH levels presents significant challenges in trying to apply findings across studies. If our current and previous results [20] are taken in conjunction with other studies on bivalve acid-base regulation [18], [80] and with the sensitivity of various life-history stages and carry-over effects [19], [62], [75], [119–121] we should be able to begin integrating these findings into an updated model of organismal, or at least bivalve, response to OA. The goal of such a model is to better understand how the complex changes in coastal carbonate chemistry due to excess dissolved carbon dioxide [2], [3], [4], [16], [122–124] may affect bivalve population dynamics through recruitment limitation via OA-stress induced larval losses. Although we cannot fully answer these questions here, our experimental techniques should allow investigators to address these and other related questions among other taxa found in coastal ecosystems where carbonate chemistry becomes more dynamic and complex.
